# HDV-Like Viruses

**DOI:** 10.3390/v13071207

**Published:** 2021-06-23

**Authors:** Jimena Pérez-Vargas, Rémi Pereira de Oliveira, Stéphanie Jacquet, Dominique Pontier, François-Loïc Cosset, Natalia Freitas

**Affiliations:** 1 CIRI—Centre International de Recherche en Infectiologie, Université de Lyon, Université Claude Bernard Lyon 1, Inserm, U1111, CNRS, UMR5308, ENS Lyon, 46 allée d’Italie, F-69007 Lyon, France; jimena.perezvargas@gmail.com (J.P.-V.); remi.pereira_de_oliveira@ens-lyon.fr (R.P.d.O.); natalia.bezerra-de-freitas@ens-lyon.fr (N.F.); 2 LBBE UMR5558 CNRS—Centre National de la Recherche Scientifique, Université de Lyon 1—48 bd du 11 Novembre 1918, 69100 Villeurbanne, France; stephanie.jacquet@univ-lyon1.fr (S.J.); Dominique.Pontier@univ-lyon1.fr (D.P.)

**Keywords:** HDV-like viruses, helper virus, origin, evolution

## Abstract

Hepatitis delta virus (HDV) is a defective human virus that lacks the ability to produce its own envelope proteins and is thus dependent on the presence of a helper virus, which provides its surface proteins to produce infectious particles. Hepatitis B virus (HBV) was so far thought to be the only helper virus described to be associated with HDV. However, recent studies showed that divergent HDV-like viruses could be detected in fishes, birds, amphibians, and invertebrates, without evidence of any HBV-like agent supporting infection. Another recent study demonstrated that HDV can be transmitted and propagated in experimental infections ex vivo and in vivo by different enveloped viruses unrelated to HBV, including hepatitis C virus (HCV) and flaviviruses such as Dengue and West Nile virus. All this new evidence, in addition to the identification of novel virus species within a large range of hosts in absence of HBV, suggests that deltaviruses may take advantage of a large spectrum of helper viruses and raises questions about HDV origins and evolution.

## 1. Introduction

Hepatitis D virus (HDV) was discovered 40 years ago in the liver of individuals chronically infected with hepatitis B virus (HBV) [1], a liver-specific pathogen present in ca. 250 million people. HDV is classified as the prototype and unique member of the *Deltavirus* genus. HDV forms enveloped particles with an average diameter of 36 nm that consists of cell-derived lipid vesicles harboring HBV surface proteins coating an inner ribonucleoprotein (RNP) [1]. This RNP is composed of a multimer of the HDV-encoded delta antigen (HDAg) [2] that is associated with one copy of a 1.7 kb long circular single strand HDV RNA exhibiting self-annealing properties [2]. Although HDAg was initially considered as a novel HBV antigen [3], it was later shown to be associated with a small RNA as a transmissible and defective agent that uses the HBV envelope glycoproteins (GP) for its propagation, hence reflecting its nature of obligate satellite of HBV [4]. For cell egress of its RNPs, HDV relies on the assistance of the helper HBV for the provision of GPs and a budding mechanism. Their envelopment subsequently allows targeting and entry of HDV particles to human hepatocytes via mechanisms that depend on the same host factors that govern the early entry events of HBV itself. Although HBV-related viruses (i.e., hepadnaviruses) are found in a number of mammals, HDV has thus far only been found in humans [1].

## 2. Current Hypotheses of HDV Origin

It is noteworthy that the origin of HDV is currently unknown. The HDV genome is unique and the smallest among animal viruses but shares some properties with some plant agents called viroids [5]. Indeed, the replication of HDV RNA involves the HDAg-mediated subversion of cellular RNA polymerase(s) (RNAP), such as Pol-II [6]. Both genomic HDV RNA and antigenomic RNA (its replication intermediate) strands include ribozyme autocatalytic, self-cleaving elements. However, most known viroids (i.e., pospiviroids) replicate by an asymmetrical rolling-circle mechanism. This pathway involves the generation of linear multimeric head-to-tail RNAs of negative polarity from circular RNAs of positive polarity by host RNAP(s). Interestingly, cells from highly divergent organisms express several HDV-like cellular ribozymes [7], which raises the possibility that HDV RNA originated from the cell transcriptome itself, in agreement with the finding that circular RNA (circRNA) species are abundant in cells [8]. The antigenomic RNA encodes for two forms of proteins based on RNA editing [9] and are referred to as small (S-HDAg, 195 amino acids) and large (L-HDAg, 214 amino acids) delta antigen [10]. S-HDAg promotes replication [11], whereas L-HDAg has been claimed to act as a dominant inhibitor of S-HDAg [12,13] and plays an essential role in virus assembly [14].

A cellular protein termed delta-interacting protein A (DIPA), initially identified as an HDAg ligand in a yeast two-hybrid screen [15], was proposed to represent a cellular ancestor of HDAg. DIPA and HDAg have shown 24% of protein sequence identity and 54% of similarity. Both proteins form oligomers through the coiled-coil domain, and it was suggested that the DIPA gene is a homolog of HDV and that capture of DIPA transcripts by a viroid-like sequence could have initiated the evolution of HDV [15].

Another hypothesis was that HDV RNA might have emerged as a consequence of an aberrant splicing event. A process known as mis-splicing, which produces RNA circles, was proposed; this occurs when a downstream (5′) splice donor is joined with an upstream (3′) splice acceptor site. However, an event of RNA-primed hairpin formation needs to occur in order to explain the base pairing of the HDV RNA [16,17].

Thus, it is theoretically possible that HDV could have emerged in HBV-infected hepatocytes subsequent to the evolution of cellular circRNA forms becoming autonomously replicative [18] and for which the ribozyme and HDAg-coding RNA sequences may have arisen from the human transcriptome [15]. That HBV, a strictly liver-tropic human pathogen, only provides RNP envelopment and transmission functions to HDV would therefore explain why HDV has been exclusively detected in the liver of HBV-infected patients. However, HDV RNA can self-replicate in a much wider variety of cell types and species [19] and this raises the possibility that HDV may be transmitted through unorthodox means. Since HDV RNA can persist in the liver in the absence of HBV for several weeks [20], it is theoretically possible that its propagation could be triggered upon superinfection by other hepatitis viruses as well as by alternative infections with HBV-unrelated viruses [21]. A central component of these hypotheses is that HDV had been found, until recently, only in humans. Yet, several viruses closely related to HDV have now been detected in non-human species in the absence of any hepadnavirus [22,23,24,25,26,27]. Further studies are warranted to ascertain whether these agents represent satellite viruses dependent on a helper virus for their propagation, or alternatively, they are examples of a novel family of autonomous viroid-like agents in animals, whose transmission would occur by conventional receptor-mediated means.

## 3. Discovery of HDV-Like Viruses

For almost 40 years, HDV formed and was the sole member of the genus Deltavirus. Recently, however, virus-prospecting studies have discovered hepatitis D relatives (known as “deltaviruses”) in a diverse range of animals (Table 1). Mining virus sequences from RNA libraries led to the discovery of two novel HDV-like sequences in ducks [22] and in Boa constrictors [23], indicating that deltaviruses likely exist in several taxa and that HDV has a far longer evolutionary history than previously anticipated. This hypothesis has been gaining ground, particularly after the identification of more deltavirus-like sequences in both vertebrates and invertebrates. Meta-transcriptomic browsing across a large set of RNA-Seq libraries from diverse animal hosts found four new HDV-like agents in a mixture of fishes (classes Actinopterygii, Chondrichthyes, and Agnatha), two amphibian species, and in termites [24]. These pioneering findings propelled more RNA-sequencing datasets searches for HDV-like sequences. Mammal deltaviruses have been identified in a neotropical rodent species *Proechimys semispinosus* (RDeV; [27]); in common vampire bats *Desmodus rotundus* (DrDeV-A and DrDeV-B); and in a liver transcriptome from a lesser dog-like bat *Peropteryx macrotis* (PmacDeV) [26]. Additional mammal deltavirus genomes were recovered from transcriptomes derived from white-tailed deer (*Odocoileus virginianus*; OvirDeV) and from captive-born Eastern woodchucks (*Marmota monax*; mmDeV) [25,26]. Three more deltavirus-like sequences have been also identified in birds, *Taeniopygia guttata* deltavirus (tgDeV), *Serinus canaria*-associated deltavirus (scDeV), and *Erythrura gouldiae* deltavirus (egDeV) [25].

In addition to the discovery of several novel deltavirus sequences in a broad host range spanning several taxonomic hosts, these metatranscriptomic approaches across different tissues and organs stressed important features of these novel deltaviruses infections; in particular, they highlighted tissue tropism differences among them and HDV. SDeV genome reads were found in NGS libraries prepared from the brain, blood, and liver [23]. Similarly, RDeV, OvirDeV, and tgDeV infections do not seem to be limited to the liver, as the virus’s sequence reads have been obtained from at least two different tissues. On occasion, data were consistent with a broad range or even systemic infection [23,25,27].

Increased viral reads from the predicted transcribed regions, i.e., delta-like antigens coding region, have been used as a marker of active deltavirus replication/transcription [25], even though HDAg mRNA is the least prevalent viral RNA species during HDV infection [28]. Indeed, with some exceptions, there is a lack of experimental proof that the newly identified HDV-like sequences are capable of self-replicating. Furthermore, there are scarce data regarding their transmission potential within the animal population and limited knowledge about the molecular mechanisms of viral infection and propagation.

Using conventional RT-PCR, SDeV was found in the liver of four out seven individuals of the offspring of the SDeV positive parental founder snakes as well in a water python that lived in close proximity. Additionally, 3 out of 20 blood samples from snakes from a different breeder turned to be positive for SDeV, suggesting that SDeV can be transmitted among animal populations [23]. However, further studies are required to ascertain whether SDeV is transmitted vertically or horizontally. In this respect, RDeV presence in the blood of adult animals only argues against vertical transmission [27]. Instead, it suggests that mammal deltaviruses similarly to HDV are passed horizontally and likely share similar routes of transmission.

Another unresolved question in the field is whether the newly reported deltaviruses can establish chronic infection like for the human HDV. Most likely the helper viruses involved and the efficacy of the host response combined shall determine the natural course of non-HDV deltaviruses infections. For tgDeV, mmDeV, and RDeV, it has been suggested that a host immune response can be mounted to suppress and clear deltavirus infections. For instance, the number tgDeV reads in birds correlated with the immunosuppressive effect of testosterone [25]. For one mmDeV positive woodchuck, the number of mmDeV genome reads over 27 weeks was compatible with acute infection [25], and the average viral load of RDeV was found to be decreased among animals with the highest antibody response against the rodent delta antigen (RDAg) [27].

Despite being highly divergent at the level of primary sequence, these non-human deltaviruses share many features with HDV. All characterized deltaviruses have a negative-sense self-complementary circular RNA genome folded into the classical unbranched rod-like structure, including ribozymes [22,25,26,27]. While the presence of ribozymes has not been reported in the four metazoan HDV-like genomes identified by [24], a more recent study confirmed the presence of self-cleaving motifs in these HDV-like genomes [29]. HDV-like ribozymes have been recently mapped in the newt and fish HDV-like sequences [29]. Contrastingly, the toad and termite HDV-like genomes lacked analogous HDV-like ribozymes and instead showed the presence of conserved type III hammerhead ribozymes, which belong to an unrelated class of catalytic RNAs characteristic of plant genomes and plant subviral circular RNAs, such as some viral satellites and viroids [29].

Nevertheless, all of the newly identified sequences include a single open reading frame (ORF) representing the delta antigen encoding for proteins with sizes ranging from 180 to 225 amino acids (DAgs). It is noteworthy that a second ORF was identified in the genome of SDeV [23], though nothing is known about its function or if it is actually expressed during SDeV replication. Important functional domains and post-translational modification sites are conserved among all DAgs of the novel deltaviruses.

The most important and intriguing observation from these virus prospecting studies, based on epidemiological and serological evidence supported by metagenomic and transcriptomic findings, is the concept that deltaviruses infections and transmission seem to be independent of coinfection with an HBV-related hepadnavirus. With the exception of mmDeV, all of the other HDV-like agents were found in the absence of hepadnaviruses [25]. In addition, the discovery of animal relatives of HDV, in particular in mammals, raises the hypothesis that hepatitis delta has jumped from a non-human animal to humans, and thus, it can be recognized as a zoonotic disease. Interestingly, all of the newly mammal deltaviruses were retrieved from hosts from the New World, the Americas, suggesting that HDV may have originated in the New World contrasting with the current hypothesis of HDV origin in Africa. Indisputably, the discovery of more mammalian deltaviruses and their hosts will help in better understanding HDV evolution, ascertaining their capacity for crossing species genetic barriers, and evaluating their potential to emerge in novel species and cause disease.

**Table 1:** Summary of Deltavirus infections.

Deltavirus	Host	CXXQ Motif	Putative Co-Infecting Viruses	Tested Helper Viruses (In Vitro)	HBsAg Usage (In Vitro)	Refs
HDV	Human	Yes	HBV	^a^ HBV,^b^ VSV, ^c^ HCV, ^d^ DENV, WNV, ^e^ LCMV, ^f^ HMPV	Yes	[21,30,31,32]
aDeV	ducks	no	Influenza A virus	nd	nd	[22]
RDeV	rodent *P.* *semispinosus*	no	Hepacivirus	nd	nd	[27]
SDeV	Boa constrictor	no	Reptarenavirus, hartmanivirus	^g^ UHV-2, UGV-1 ^h^ HISV-1 ^e^ LCMV, JUNV ^f^ PUUV	no	[23,33]
tgDeV	Zebra finch *Taeniopygia guttata*	no	none	nd	no	[25]
mmDeV	Eastern woodchuck *Marmota monax*	no	WHV, herpesvirus, flavivirus, retrovirus	nd	no	[25,26]
DrDeV	Vampire bats *D. rotundus*	no	Herpesvirus, flavivirus, retrovirus	nd	nd	[26]
OvirDeV	White-tailed deer *Odocoileus* *virginianus*	no	Herpesvirus, flavivirus, retrovirus	nd	nd	[25,26]
PmacDeV	Lesser dog-like bat *Peropteryx macrotis*	no	Herpesvirus, flavivirus, retrovirus	nd	nd	[26]

List of newly identified deltaviruses and their hosts. Presence of the CXXQ motif in the virus antigen is indicated. The potential enveloped helper viruses based on either RNA-seq data, epidemiological or serological data are shown. Additionally, listed are the candidate helper viruses that were shown to successfully support deltaviruses assembly and infectivity in vitro. HBsAg usage, whenever known is indicated. ^a^ Hepadnavirues hepatitis B virus (HBV), woodchuck hepatitis virus (WHV), tent-making bat HBV (TBHBV) and Woolly monkey HBV (WMHBV); ^b^ Vesiculovirus vesicular stomatitis virus (VSV); ^c^ Hepacivirus hepatitis C virus (HCV); ^d^ Flaviviruses dengue virus (DENV) and West Nile virus (WNV); ^e^ Mammarenaviruses *Lymphocytic choriomeningitis* virus (LCMV) and Junin virus (JUNV); ^f^ Orthohantaviruses human metapneumovirus (HMPV) and Puumala virus (PUUV); ^g^ Reptarenaviruses UHV-2 and UGV-1; ^h^ Hartmanivirus HISV-1; nd-nondetermined.

## 4. Phylogeny

Based on the percentage of nucleotide identity of the genome, HDV was classified into eight genotypes, with two to four subtypes per genotype characterized by >90% similarity over the entire genome sequence [34,35,36]. The distribution of various HDV genotypes was associated with geographical origins; however, the geographical distribution has changed over time, probably due to human migration patterns. Between genotypes, there can be as much as a 35% difference in nucleotide sequence [35].

The discovery of divergent HDV-like viruses from different hosts raises questions about the origin and evolution of HDV. Consequently, phylogenetic inferences and sequence analysis have been conducted each time a new deltavirus species was discovered. One of the first phylogenetic analyses estimated an average nucleotide identity of 29 to 46% and amino acid identity of 21 to 54% among all HDV genotypes and 7 of the newly reported deltaviruses [27]. RDeV was the single nonhuman mammalian deltavirus included in this study, and thus, not surprisingly, the phylogenetic reconstruction identified RDeV as the nonhuman deltavirus most closely related to human HDV clustering in the same clade with SDeV [27].

**Figure 1:**
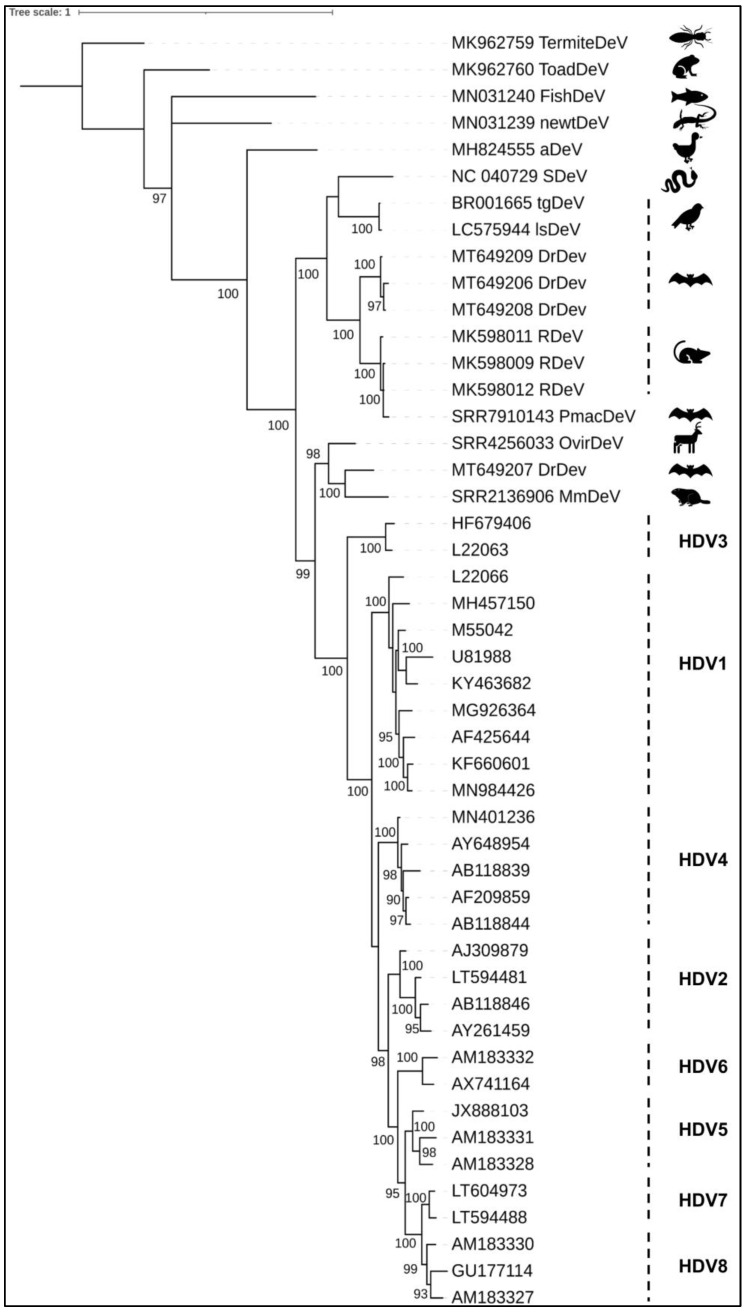
Bayesian phylogenetic tree of mammalian deltaviruses and HDV-like viruses. Small delta antigen (DAg) nucleotide sequences from published mammalian and invertebrate deltaviruses, including representative HDV clades, were aligned using the translated protein option (without the trust insertion -F) implemented in PRANK [37,38]. Phylogenetic reconstruction of DAg was carried out using the resulting protein alignment and the best amino acid substitution model (LG+F+G) inferred by Smart Model Selection [39]. The Bayesian analysis was carried out with MrBayes, using 2,000,000 generations with a tree sampling every 1000 generations and a burn-in of 500 trees. The final tree was rooted with the termite HDV-like virus. Tree edition was performed with iTOL [40].

More recent phylogenetic analyses based on full-length protein sequences of DAg, including all HDV genotypes and the 12 HDV-like viruses described so far, revealed, however, different relationships among deltaviruses [25,26]. The mammalian deltaviruses DrDeV-A, OvirDeV, and mmDeV share a common ancestor closely related to HDV (Figure 1), whereas RDeV is genetically related to PmacDeV clustering with DrDeV-B and a clade consisting of SDeV, tgDeV, and lsDeV [26]. Other related viruses (fish, amphibian and invertebrate HDV-like viruses) constitute basal lineages of the tree (Figure 1). Indeed, these latter viruses, which display the lowest degrees of amino acid sequence identity among deltaviruses [25], constitute divergent phylogenetic lineages being the most distant from other known deltaviruses. It is noteworthy that the basal nodes of the tree are not strongly supported, suggesting that other related deltaviruses (yet to be discovered) are likely missing. The current limited number of deltavirus species also prevents from drawing definitive conclusions of the origin of HDV.

With the discovery of new HDV-like viruses, new phylogenetic analyzes will allow a better understanding and reconstruction of the evolutionary history of animal deltaviruses and their relationships with their respective hosts.

## 5. Virus Replication, Genome Editing, and Viral Proteins Expression

HDV virion encloses a circular, single-stranded negative-sense RNA molecule associated with an internal core delta antigen surrounded by envelope glycoproteins. The full-length circular genome for HDV is approximately 1680 nucleotides, whereas for HDV-like agents, it ranges between 1592 and 1735 nucleotides. The GC contents of these novel HDV-like agents range between 46 and 58 percent, which is slightly lower than the human HDV with a GC content of around 60 percent [22,23,24,25,26]. The circular nature and self-annealing properties of HDV RNAs are reminiscent of some plant viroids and are important for both genome replication and RNP assembly upon interaction with HDAg proteins [41,42,43]. Most RNA viruses encode their own replicases or RNA-dependent RNA polymerases (RdRp) essential for viral genome replication. However, HDV does not carry an RdRp gene to replicate its genome. It rather makes use of cellular DNA-dependent RNA polymerases to replicate its genome. Since HDV genomic RNA has a negative or anti-messenger polarity, during replication, three different forms of RNA are made: linear 0.8 Kb messenger RNA (mRNA) of antigenomic polarity harboring a 5′-cap and a 3′-polyadenylated tail [28,44], circular genomic RNA, and circular complementary antigenomic RNA, which are synthetized via a rolling-circle mechanism similar to that proposed for some viroids [45,46]. The rolling-circle mechanism involves unidirectional replication of nucleic acids to form multiple copies of the circular genome using cellular RNA polymerases. The process is not completely understood, but only the genomic RNA is assembled into HDV particles, even if both genomic and antigenomic RNA replication is detected in the nucleus, suggesting that there is a selective export of genomic RNA to the cytoplasm [47]. Nevertheless, in vitro antigenomic RNA can be secreted as long as the large hepatitis delta antigen, L-HDAg, and HBsAg are coexpressed [48].

A ribozyme, a self-cleaving RNA sequence, resides in the viroid-like sequence of the HDV genome that cleaves a linear form of multiple-copy length of the viral genome or antigenome into monomeric units that are then circularized to complete the replication cycle. All described HDV-like agents contain self-cleaving motifs from either the HDV or the hammerhead class [22,23,26,27,29]. In vitro transcription and self-cleavage analysis confirmed the cleaving activity for these motifs in the newt, fish, toad, and termite HDV-like RNAs [29]. The cleaving activity for the other identified ribozymes has not yet been tested.

Many cell culture model systems have been developed to study HDV genome replication and regulation of viral gene expression. One of the most simplified systems relies on the transfection of cultured cell lines with larger than unit-length cDNA clones (usually dimers or trimers) of HDV sequences [11]. HDV cDNA vectors have been constructed to produce either genomic or antigenomic RNA and both kinds of recombinants have been shown to be competent to initiate genome replication [11,49]. In these transfection assays, the first round of HDV RNA synthesis is DNA-directed under the control of a heterologous promoter, followed by subsequent rounds of RNA-directed RNA replication that is absolutely dependent on S-HDAg [6]. RNA-directed RNA replication can be easily assayed by Northern analysis with a probe specific for the product of replication, i.e., viral RNA of opposite polarity to the RNA transcribed from the cDNA template, and viral gene expression by Western blot and immunofluorescence [11,49].

Using an analog approach, it was shown that RDeV could initiate replication in Huh7 cells transfected with a construct carrying a head-to-tail dimer of the full RDeV genome in genomic orientation as demonstrated by the presence of the marker of on-going RNA-directed RNA replication, i.e., viral RNA of antigenomic polarity and S-RDAg as early as 4 days post-transfection [27].

**Figure 2:**
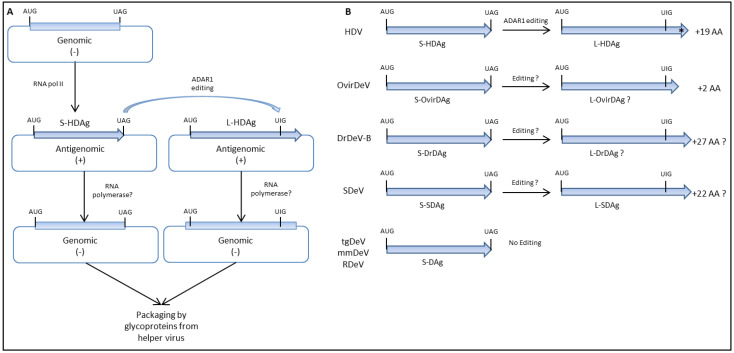
Schematic representation of RNA editing during deltaviruses replication (**A**) and putative L-DAgs generated via ADAR1 (**B**). The incoming viral genome, a circular ssRNA molecule of negative polarity (genomic RNA), serves as a template for the synthesis of DAg encoding mRNAs and circular RNAs of antigenomic polarity. The mRNA produced at the early stages of deltaviruses replication contains an amber stop codon (UAG) and produces the small form of DAgs. In a fraction of the antigenomic RNAs, the adenosine in the amber stop codon is deaminated to inosine (UIG) by the host RNA editing enzyme ADAR1. The edited mRNAs contain a tryptophan (W) codon instead of the amber stop codon and additional codons are translated to yield the large form of DAgs. The asterisk depicts the presence of a CXXQ motif in the C-terminus of L-HDAg required for isoprenylation.

HDV uses RNA editing as a mechanism to switch from genome replication to packaging. During replication, a fraction of the antigenome RNA is edited by ADAR1 such that the adenosine within the amber stop codon that terminates the S-HDAg ORF is deaminated to inosine (Figure 2). The stop codon is thus changed to tryptophan and the ORF is extended by additional 19 or 20 amino acids to yield L-HDAg. Within the 19 C-terminal amino acids unique to L-HDAg, a nuclear export signal (NES) has been identified [50]. It also contains a conserved CXXQ box motif that is post-translationally modified with an isoprenyl anchor [51]. These lipid moieties, a farnesyl in the case of L-HDAg, are typically involved in mediating protein–membrane and protein–protein interactions. Indeed, farnesylation of L-HDAg enables the interaction between HDV RNPs and tryptophan residues of the small cytosolic loop of HBsAgs [52] and thus is required for virion assembly. It is also implicated in the mechanism by which L-HDAg inhibits HDV RNA replication [53]. Interestingly, farnesyl-mediated targeting to cellular membranes of L-HDAg seems to assist HDV assembly with envelope proteins from unorthodox helper viruses [21].

Similar to S-HDAg, the RDAg ORF also terminates with a UAG amber stop codon [27]. A similar A-to-I editing of the RDeV antigenome RNA could eliminate the stop codon of the S-RDAg ORF and extend the ORF by additional 19 amino acids. In Huh7 cells transfected with HDV cDNAs, L-HDAg levels are barely detectable during the first post-transfection days but then progressively increase relative to S-HDAg [43]. Western blot analysis showed no signs of a large RDAg antigen (L-RDAg) expression in Huh7 cells transfected with a dimeric RDeV cDNA construct monitored every 2 days for a total period of 10 days [27]. Consistently, there was also no evidence of RNA editing using RNA-seq data of the blood and organ samples of infected animals and RNA sequences extracted from the transfected cells. Overall, it seems that during RDeV infection, antigenomic editing and L-RDAg production do not occur [27].

In contrast to RDeV, two species of NGS reads were detected for OvirDeV at the second position of the stop of the S-OvirDAg (UAG). The base-editing frequency measured was 0.4% with all edited sequences showing a G instead of the consensus A. While this A-to-I nucleotide variation may suggest RNA editing by ADAR1, the generated ORF would be extended by two amino acids only due to the presence of a stop codon immediately after [25]. However, there are no experimental data addressing OvirDeV editing or large antigen expression.

A putative 28-amino acid carboxyl-terminal extension was also identified in the bat deltavirus (DrDeV) genome [26], although there is no experimental evidence of DrDeV RNA editing or large antigen expression. Even though RNA editing could occur, the putative resultant protein, as well as all other deltaviruses putative large antigens lack a farnesylation motif. For mmDeV and tgDeV, no nucleotide variations could be identified among the stop codons of the viral genes, suggesting that these two viruses do not undergo RNA editing during replication [25]. A single form of the virus antigen was detected after transfection with mmDeV and tgDeV replication competent cDNA constructs [25].

Sequence analysis of the SDeV genome revealed a potential editing site at the UAG amber stop codon of the S-SDAg. RNA editing would increase the ORF by 22 additional amino acids, and thus, two bands with estimated molecular weights of 22.7 kDa and 25.6 kDa should be detected by Western blotting in the homogenates of tissue samples from infected snakes. Indeed, an anti-S-SDAg antiserum detected two bands with molecular weights that are approximate to the theoretical estimates for S- and L-SDAg in liver samples of infected snakes but mainly only the large form was detected in the brain samples [23]. Whether the two bands observed are tissue dependent and result from RNA editing still remains unclear because the study does not provide NGS data in support of antigenomic virus RNA editing. Additional studies are thus awaited to address the differences in SDAg processing and maturation. Another peculiarity concerning SDAg is its prominent cytoplasmic staining despite the presence of putative nuclear localization signals (NLSs) in its amino acid sequence in different tissues of infected Boa constrictor snakes [23]. SDAg distribution pattern was further analyzed by transfection of boid and monkey kidney cell lines, I/1Ki and Vero E6 cells, respectively. In both cell lines, SDAg was predominantly found in the cytoplasm independently of the orientation of the SDeV cDNA used to initiate virus replication [33]. Similar results were obtained in human kidney and cervical HeLa cells and in snake lung cells. However, a nuclear staining resembling HDAg was also found in human lung cells as well as in snake and human heart and liver cell lines.

In contrast to HDV, RNA editing seems not to be the mechanism regulating the switch from replication to viral RNPs packaging for the newly identified deltaviruses (Figure 2). Additionally, the lack of large antigen expression and farnesylation should prevent the interaction with hepadnaviral envelope proteins.

HDV RNA and HDAg proteins interact to form the HDV ribonucleoprotein (RNP), the delta antigens form multimers, and the HDV RNA wraps around such multimers. It has been suggested that the multimers may be predominantly octamers of the delta antigen and that the genomic RNA, which is partially double-stranded, wraps around the octamers in a way analogous to how double-stranded host DNA wraps around histone octamers [2,54]. The predicted coiled-coil domains of HDAgs that facilitate multimerization and replication, found at the N-terminal region between amino acids 22 and 44, were also found in HDV-like agents [22,27].

## 6. Helper Virus’s Functions

The envelope proteins of HBV are the only contribution of the helper virus to the HDV life cycle. The determinants governing HBV tissue and species tropism also dictate HDV tropism to human hepatocytes. HBV and HDV entry requires the specific interaction with the preS1 domain of viral L-HBsAg. Despite sharing the same envelope and using the same host receptor, HBV and HDV show different requirements of host factors to establish productive infections. In contrast to HBV, HDV infection is predominantly determined at the receptor level [55]. Ectopic expression of human NTCP is enough to confer cells from different tissue and species susceptibility for HDV, whereas additional host factors besides NTCP are required for successful HBV infection [55,56]. HDV RNA replication is entirely independent of the helper virus. Indeed, HDV RNA can persist in the liver of humanized mice in the absence of HBV for several weeks and potentially be rescued by a later HBV infection [20]. Additionally, primary Sjögren’s syndrome patients were reported to present HDV antigen and RNA in salivary glands in the absence of HBsAg or HBV antibodies [57].

For budding HDV takes advantage of the quite inefficient HBV assembly process that produces relatively huge amounts of empty particles [58]. The budding process involves interaction between the farnesylated Cys-211 of L-HDAg with the cytosolic loop of the HBsAg, and it can be independent of active HBV replication within the same cell. In line with this view, infectious HDV particles can be produced in cells lacking markers of ongoing HBV replication but expressing HBV envelope proteins from integrated virus DNA into the host cell genome [59]. Experimentally, HDV can also make use of envelope proteins of WHV to produce infectious progeny. These particles were shown to be capable of infecting both primary human hepatocytes (PHH) and woodchuck primary hepatocytes (WPH), whereas HDV assembled with HBV cannot enter WPH [32]. Recently, we demonstrated that HDV can be transmitted and propagated in experimental ex vivo and in vivo infections by different enveloped viruses unrelated to HBV, including hepatitis C virus (HCV), flaviviruses such as Dengue and West Nile viruses, and Vesiculovirus [21].

Similar to HDV, the newly identified deltaviruses RNA can self-replicate in a wide variety of cell types and species [19,23,27,33], indicating that while deltavirus replication is not limited to any particular cell type, it is restricted by the envelope glycoproteins borrowed from the helper virus. As for HDV, mmDeV and SDeV cDNA transfection can lead to a persistent infection that survives cell division without the requirement of a helper virus [25,33]. While there is no information available describing the competence of snake cell lines supporting HDV replication, SDeV persistence lasted up to 6 months post-transfection, at least two times longer than HDV in Huh7 cells [60]. This persistence seems to be cell type and/or species dependent since SDeV could not establish persistent infection in human cells, suggesting the existence of virus-intrinsic and/or host-specific determinants determining deltavirus replication. Even though direct cell–cell SDeV spread cannot be excluded without control experiments, it seems that SDeV persistence results from clonal expansion, rather than cell-free virus transmission as the supernatants from the chronically infected snake cells were incapable of passing SDeV infection to naïve cells [33].

While the potential candidate helper viruses for these novel deltaviruses are currently unknown, the lack of a farnesylation motif (CXXQ), required for HDV assembly and release in the C-terminal region of the novel deltaviruses antigens (Table 1), highlights new and alternative mechanisms for deltaviruses envelopment and cell egress [25,27].

## 7. Candidate Helper Viruses

HDV transmission among the human population requires HBV envelope proteins. Likely, the transmission of the newly reported deltaviruses in animal populations might also be dependent on coinfecting helper viruses to provide envelope proteins to copackage deltaviruses RNPs and assist dissemination.

The final stage of the HDV life cycle, that is, the incorporation of the newly replicated RNA genome associated with delta antigens into enveloped virions, can be easily accomplished by coinfection with a helper virus or by cotransfection with constructs encoding the envelope proteins of hepadnavirus belonging to the genus *Orthohepadnavirus*.

For decades, only two hepadnavirus genera were known: genus *Orthohepadnavirus*, which infects mammalian species, and genus *Avihepadnavirus*, which infects avian species. Since 2019, however, five hepadnavirus genera are recognized [61]. The three newly defined genera include the herpetohepadnaviruses (genus *Herpetohepadnavirus*), which infect amphibians and reptiles, as well as two highly distinct groups that infect fishes—the metahepadnaviruses (genus *Metahepadnavirus*) and the parahepadnaviruses (genus *Parahepadnavirus*) [62,63,64].

Since HDV has to coexist with HBV for propagation, a first assumption would be that HDV should have evolved in animals that were susceptible to infection by hepadnaviruses, namely, two of the newly discovered deltaviruses, aDeV and mmDeV, were retrieved from RNA-seq libraries of ducks and eastern woodchucks that are natural hosts for hepadnaviruses [22,25] and can be coinfected with their respective Orthohepadnavirus species, the duck hepatitis B virus (DHBV) and the woodchuck hepatitis virus (WHV).

SDeV isolated from brain homogenates was shown to be able to establish persistent infection in boid kidney cells (I/1Ki cells). The same metatranscriptomic analyses that led to SDeV discovery revealed that the infected snakes had no traces of coinfection with HBV-like viruses. Instead, NGS analyses retrieved sequences of the L and S RNA segments of reptarena- and hartmanviruses [33], suggesting that arenaviruses could act as SDeV helper viruses and provide the envelope glycoproteins required for the formation of infectious particles. Indeed, staining of SDeV infected cells with antibodies against SDAg and anti-, reptarena-, or hartmanviruses nucleoprotein (NP) suggested the SDeV-brain homogenate to be coinfected with all three viruses. In vitro superinfection of permanently SDev-infected snake cells with reptarenaviruses (UHV-2 and UGV-1), and the hartmanvirus HISV-1 confirmed that SDeV could use arenaviruses as helper viruses to produce SDeV infectious particles capable of infecting naïve cells. Furthermore, it was demonstrated that transfection of plasmids encoding the glycoproteins of HISV-1, UGV-1, the othohantavirus Puumala virus (PUUV), the lymphocytic choriomeningitis virus (LCMV), and Junin virus (JUNV) was enough to drive the production of SDeV infectious particles but not a synthetic gene covering the L, M and S surface proteins of HBV. However, the infectivity of SDeV particles pseudotyped with HBV glycoproteins was assessed on snake kidney cells, an unsuitable cell culture model to evaluate HBV-envelope-mediated entry, which has limited cellular and host tropism [33].

Many in vitro and in vivo infection studies demonstrated that woodchuck hepatitis virus (WHV), a hepadnavirus related to HBV, could provide helper activity for HDV [32,65]. As one of the newly identified deltaviruses, mmDeV was identified in the liver transcriptome from Eastern woodchucks (*Marmota monax*) experimentally infected with WHV and in two sequence read archive (SRA) data derived from PMBCs of animals negative for antibodies against WHV as well as WHV DNA [25]. The liver tropism and coinfection with mmDeV and WHV could suggest a well-established satellite/helper relationship between another mammalian deltavirus and a hepadnavirus. However, WHV was experimentally inoculated in these animals, and therefore, the presence of both viruses in the liver may not reflect important virus association for mmDeV transmission. Additionally, mmDeV does not seem to utilize the A-to-I RNA editing mechanism to extend the viral ORF and neither carries a CXXQ signal required for farnesylation [25]. In line with the idea that hepadnaviruses do not provide helper activity for mmDeV transmission, it was demonstrated that HBsAgs were not capable of supporting mmDeV or tgDeV assembly and infectivity [25].

Despite the lack of experimental evidence, the libraries dominated with WHV also contained reads matching viral sequences of Herpesvirus, Flavivirus, Retrovirus, and Poxvirus, representing potential sources of helper activity. Interestingly, Poxvirus hits were identified in libraries where four other deltaviruses have been identified, i.e., DrDeV-A, DrDeV-B, PmacDeV, and OvirDeV, which was hence considered as a potential ecological association worth to be evaluated in future studies [26].

As hepacivirus infections are highly prevalent in rodents and since HCV, in particular, can provide envelope proteins for HDV and support its transmission in vivo [17,21], the question of whether hepacivirus could support RDeV infection has been raised. Although 26 out of the 30 RDeV RNA-positive rodents were also positive for hepacivirus, statistical analyses did not recognize any significant association between RDeV and hepacivirus infections in the 763 animals sampled [27]. Whereas the suggested lack of dependency between RDeV and hepacivirus infections can be explained by the absence of a farnesylation motif in the rodent delta antigen, the possibility of alternative enveloping mechanisms, enabling the usage of hepacivirus envelope proteins by RDeV, remains to be investigated. A noteworthy finding is that both HDV and SDeV can be assembled with Hantaviruses and Arenaviruses envelope proteins strengthens this hypothesis [21,33]. It suggests that even HDV RNPs incorporation into enveloped particles can be independent of host-mediated prenylation of L-HDAg and also implies that divergent deltaviruses could share alternative-enveloping mechanisms. Future research studies will certainly address these and other questions. For instance, do retroviruses support non-human deltaviruses assembly and infectivity contrasting with their inability to sustain HDV propagation [21]?

## 8. Conclusions

Undoubtedly, the discovery of HDV-like viruses in distantly related species other than humans and their detection in the absence of HBV or hepadnavirus infection, as well as the evidence that HDV and HDV-like virus can indeed use envelope glycoproteins from viruses of diverse genera for transmission, open numerous questions over the origin of deltaviruses and the nature of the relationship between HDV and HBV. This suggests that HDV-like viruses have been associated with animals long before their first appearance in humans [36] and during their evolutionary history. It is still possible that the ancestor of deltaviruses emerged from cellular RNAs; however, it seems likely that it would have happened much earlier in evolution than previously thought. However, we cannot discard the hypothesis that a common ancestor of deltaviruses could be found among viroids of plants, as they show the highest similarities with deltaviruses [66,67]. Since a deltavirus has already been identified in termites [24], one possibility is that the deltavirus ancestor was transmitted to animals from plants, since the presence of viroids in aphids feeding on infected plants was already shown [68], which could support the above hypothesis. The phylogeny suggests that the mammalian deltaviruses are closer to HDV common ancestor, whereas avian, reptile, fish, or amphibian HDV-like viruses are more distant. Nevertheless, the taxonomic distance among the deltavirus hosts does not fully support the hypothesis of virus–host codivergence. Based on the phylogenetic tree, it rather supports cross-species transmissions in mammals. The discovery of HDV-like viruses in other species is necessary to have more elements to answer the questions regarding the evolution of deltaviruses. It is also tempting to speculate that deltaviruses had an origin in other species different than humans and use other helper viruses than HBV-related viruses; yet, in humans, HDV may have found HBV as its best partner.

It is noteworthy that three clinical studies have now been performed to verify the possibility of HDV infection in patients only coinfected with HCV [69,70,71]. Although one study from our group provided preliminary evidence of HDV exposure in chronically HCV-infected patients in the absence of ongoing or past HBV infection [70], overall, these studies also highlighted the rarity of HDV and HCV coinfection events at least in such patients.

Altogether, these studies pave the way to a better understanding of the origin of HDV and enlarge the deltaviruses research field. They also raise several questions concerning the immunological and virologic relations between HDV and HBV or alternative helper viruses, and the exact pathogenesis and tissue tropism of HDV. Perhaps one of the most important questions is if HDV can be transmitted in humans by other helper viruses than HBV. Additionally, if HDV has a wider range of potential coinfections than previously thought, what could be the clinical relevance and pathobiological implications?
